# A PTPmu Biomarker is Associated with Increased Survival in Gliomas

**DOI:** 10.3390/ijms20102372

**Published:** 2019-05-14

**Authors:** Mette L. Johansen, Jason Vincent, Haley Gittleman, Sonya E. L. Craig, Marta Couce, Andrew E. Sloan, Jill S. Barnholtz-Sloan, Susann M. Brady-Kalnay

**Affiliations:** 1 Department of Molecular Biology and Microbiology, School of Medicine, Case Western Reserve University, 10900 Euclid Ave, Cleveland, OH 44106-4960, USA; mette.johansen@case.edu (M.L.J.); jason.vincent@case.edu (J.V.); sonya.ensslen@case.edu (S.E.L.C.); 2 Department of Population and Quantitative Health Sciences, School of Medicine, Case Western Reserve University, 10900 Euclid Ave, Cleveland, OH 44106, USA; haley.gittleman@case.edu (H.G.); jill.barnholtz-sloan@case.edu (J.S.B.-S.); 3 Department of Neurological Surgery, University Hospitals of Cleveland, Seidman Cancer Center and Case Comprehensive Cancer Center, School of Medicine, Case Western Reserve University, 10900 Euclid Ave, Cleveland, OH 44106, USA; marta.couce@uhhospitals.org (M.C.); andrew.sloan@uhhospitals.org (A.E.S.); 4 Department of Neurosciences, School of Medicine, Case Western Reserve University, 10900 Euclid Ave, Cleveland, OH 44106, USA

**Keywords:** receptor type protein tyrosine phosphatase PTPmu, glioblastoma, glioma, biomarker, adolescent and young adult, molecular analysis of glioma

## Abstract

An integrated approach has been adopted by the World Health Organization (WHO) for diagnosing brain tumors. This approach relies on the molecular characterization of biopsied tissue in conjunction with standard histology. Diffuse gliomas (grade II to grade IV malignant brain tumors) have a wide range in overall survival, from months for the worst cases of glioblastoma (GBM) to years for lower grade astrocytic and oligodendroglial tumors. We previously identified a change in the cell adhesion molecule PTPmu in brain tumors that results in the generation of proteolytic fragments. We developed agents to detect this cell surface-associated biomarker of the tumor microenvironment. In the current study, we evaluated the PTPmu biomarker in tissue microarrays and individual tumor samples of adolescent and young adult (*n* = 25) and adult (*n* = 69) glioma populations using a fluorescent histochemical reagent, SBK4-TR, that recognizes the PTPmu biomarker. We correlated staining with clinical data and found that high levels of the PTPmu biomarker correlate with increased survival of glioma patients, including those with GBM. Patients with high PTPmu live for 48 months on average, whereas PTPmu low patients live only 22 months. PTPmu high staining indicates a doubling of patient survival. Use of the agent to detect the PTPmu biomarker would allow differentiation of glioma patients with distinct survival outcomes and would complement current molecular approaches used in glioma prognosis.

## 1. Introduction

Diffuse gliomas are malignant brain tumors that consist of astrocytomas, oligodendrogliomas, and glioblastomas (GBM). They represent approximately 70% of all malignant brain tumors [1] and are categorized into either low or high grade. Low grade tumors include World Health Organization (WHO) grade II astrocytomas and oligodendrogliomas. High grade tumors include WHO grade III astrocytomas and oligodendrogliomas, as well as GBM (WHO grade IV). Low grade diffuse gliomas have the best prognosis with an overall survival of 11 years [2], whereas the overall survival for the most aggressive glioma, type IV GBM, is only 15 months [3]. In addition to tumor grade, other important prognostic factors include age at diagnosis and gender [4,5,6]. Females and younger age at the time of diagnosis are associated with longer survival in glioma patients.

Treatment of low grade glioma generally consists of debulking surgery, radiation therapy, and sometimes adjuvant chemotherapy [7]. Treatment of grade III and IV gliomas consists of surgical resection followed by radiation and chemotherapy [3], with the extent of surgical resection being a major predictor of disease outcome [8]. Conventional surgery has relied heavily upon the neurosurgeon’s professional experience to recognize tumor from normal brain tissue; now, more sophisticated approaches using magnetic resonance imaging (MRI) and/or fluorescent agents to identify tumor tissue are in use or in development [9]. Currently, one fluorescent agent, 5-aminolevulinic acid (5-ALA), is Food and Drug Administration (FDA) approved to aid in the surgical resection of high grade glioma. It is a non-specific agent that is metabolized preferentially but not exclusively in glioma tissue, and while helpful at improving the extent of surgical resection [10,11], it lacks specificity in identifying tumor margins [10,12,13].

We are interested in the roles that changes in cell adhesion play in brain tumor progression. In normal cells, the full-length receptor protein tyrosine phosphatase PTPµ mediates cell-cell adhesion through homophilic binding [14]. We discovered that full-length PTPµ is proteolyzed into fragments in GBM [15,16], whereas differential levels of full-length PTPµ are expressed in low grade astrocytoma tissue [17]. The PTPµ extracellular fragment generates a biomarker in the tumor microenvironment that can be utilized for specific molecular recognition of cancer [18]. We identified peptides, known as the SBK peptides, derived from the extracellular portion of PTPµ that bind homophilically to the extracellular PTPµ biomarker in vitro and/or in vivo [18]. Conjugating these SBK peptides to fluorophores created targeted PTPµ biomarker-directed agents that specifically bind tumor cells, including GBM, but not normal tissues [18]. Remarkably, use of these fluorescent agents in vivo with brain tumor models revealed their ability to detect the primary tumor and glioma cells that had migrated several millimeters away from the main tumor mass [19]. We have proposed developing these agents as tools for fluorescence-guided surgical resection of GBM [9]. The utility of SBK-targeted agents for imaging gliomas has also been demonstrated using preclinical MRI. For that application, the SBK peptide was conjugated to the macrocyclic molecule, 1,4,7,10-Tetraazacyclododecane-1,4,7,10-tetraacetic acid (DOTA), and complexed with gadolinium [20,21]. PTPµ biomarker targeted MRI agents showed more sustained binding to and enhancement of tumors compared to untargeted, conventional gadolinium-containing contrast agents in brain tumor models [20,21] and might be advantageous in standard MRI or intraoperative MRI.

The WHO Classification of Tumors of the Central Nervous System introduced a new “integrated” scheme using molecular markers alongside traditional histopathology to classify diffuse gliomas [22]. These recommendations include tests for mutated isocitrate dehydrogenase 1 (IDH1) status to differentiate tumors. Presence of IDH1 mutation correlates with more favorable patient survival outcomes [22]. In the present study, we characterized the staining of the PTPµ biomarker in human glioma tissue microarrays or in individual tumor biopsy samples using the SBK4 peptide conjugated to Texas Red, SBK4-TR, and correlated this with clinical and pathologic features, including survival outcomes. Our findings indicate that PTPµ high biomarker levels are predictive of longer survival time for all glioma subtypes. Even when adjusted for age, sex, and IDH1 mutation status, PTPµ high biomarker levels correlate with increased survival in GBM patients. These data provide evidence that the PTPµ biomarker may predict survival for various gliomas and support the use of the SBK agents for prognosis and imaging.

## 2. Results

### 2.1. Staining of Glioma Sections with the PTPµ Biomarker

Human glioma tissue microarrays (TMAs) or individual glioma tumor samples were obtained from 94 patients including 25 adolescent and young adult (AYA) and 69 adults with astrocytomas (*n* = 12), oligodendroglioma (*n* = 14), oligoastrocytoma (*n* = 7), and GBM (*n* = 61). The clinicopathological characteristics of the patients combined with PTPµ biomarker staining results are summarized in Table 1. The 94 patients were fairly equally divided between those with PTPµ low (52%) and those with PTPµ high biomarker levels (48%; Table 1). Significantly more patients with PTPµ high were alive at the end of the follow-up period (22 patients) compared to those with PTPµ low biomarker levels (eight patients, *p* < 0.002). These PTPµ high biomarker patients also had a significantly longer mean overall survival time of 48 months compared to the mean overall survival time of 22.4 months for the PTPµ low patients (*p* < 0.001). Survival times shown in Table 1 represent mean survival times for all patients in a group, both for those where death was recorded and for those alive at the conclusion of the follow-up period. The mean time to recurrence shown in Table 1 for each group was calculated only from patients who experienced a recurrence.

**Figure 1:**
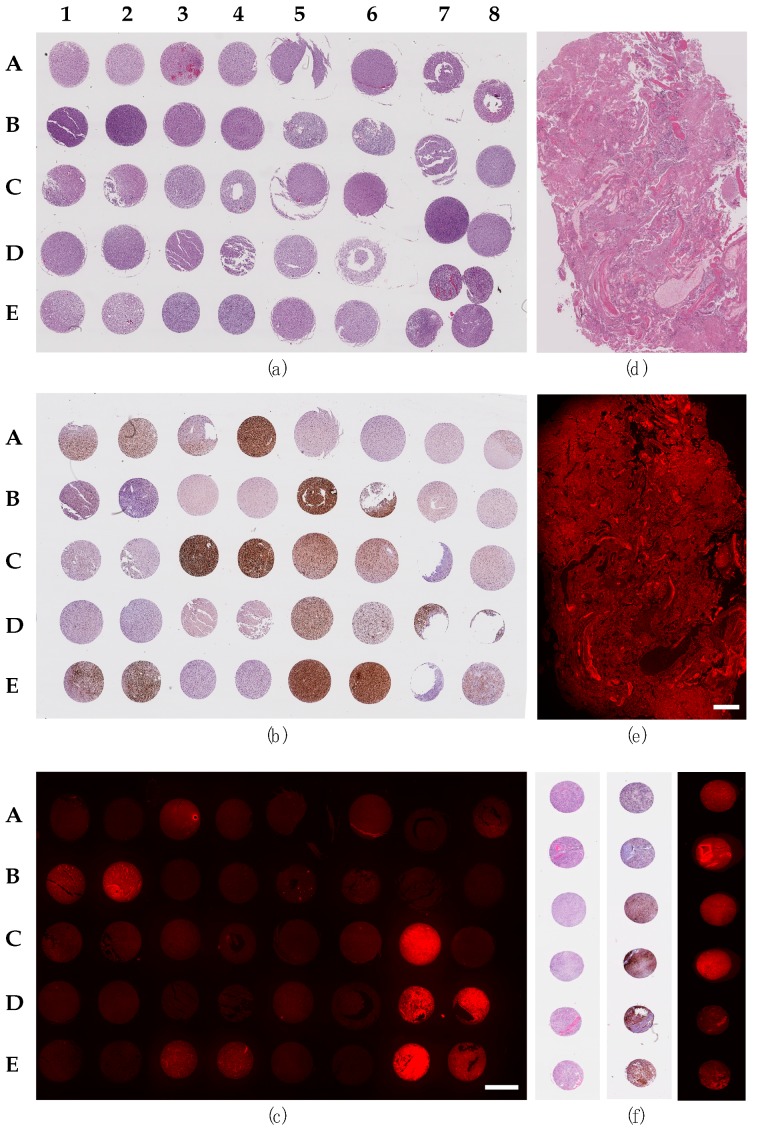
Staining for the PTPµ biomarker and mutant IDH1. These are representative examples of a TMA (**a**–**c**,**f**) or an individual slide (**d**–**e**) stained with the relevant markers. The patient samples are marked in rows A–E with numbers at the top from 1–8. Therefore, the tumor core location is referred to as A1, A2, etc. (**a**) Hematoxylin and eosin (H&E) stain of the TMA. (**b**) Mutant IDH1 staining of the TMA. (**c**) SBK4-TR stain of the TMA. (**d**) H&E of individual sample of GBM. (**e**) SBK4-TR stain of the same individual. Panel (**f**) shows results from a different TMA with H&E (left), mutant IDH1 (middle) and SBK4-TR (right) staining with a range of PTPµ high to PTPµ low and both wild-type and mutant IDH1. Scale bar = 1mm.

The samples were stained for PTPµ with SBK4-TR, and a subset of those patient samples are shown in Figure 1. Histology was visualized by staining with hematoxylin and eosin (H&E; Figure 1a,d,f). The SBK4-TR staining was visualized with a fluorescent microscope. There was variable PTPµ staining of the tumor samples (Figure 1c,e,f). The amount of fluorescence was divided into two categories, PTPµ low and PTPµ high, to reflect the biphasic nature of the results. As examples, A1 and A2 were classified as PTPµ low, while E7 and E8 illustrate PTPµ high expressing samples (Figure 1c). The TMAs were also stained for mutant IDH1, as shown in Figure 1b,f, and scored as positive or negative to replicate scoring by pathologists. A different TMA is shown in Figure 1f with samples illustrating the range of PTPµ low and PTPµ high as well as wild-type and mutant IDH1 samples.

### 2.2. Analysis of Clinical Variables in Comparison to the PTPµ Biomarker

**Figure 2:**
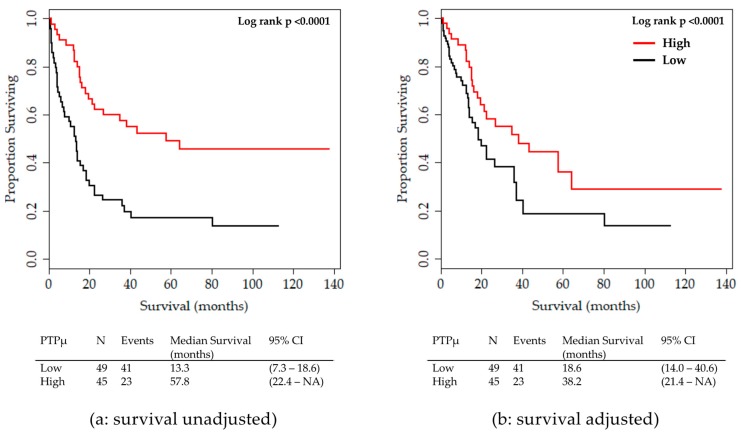
Kaplan Meier Plot for overall survival by PTPµ high versus low staining for all glioma patients. (**a**) Unadjusted overall survival. (**b**) Overall survival adjusted by sex, tumor grade, age group, and IDH1 mutation status. Median survival with 95% Confidence Intervals for each group are shown below each plot.

Kaplan Meier survival plots demonstrate that patients with PTPµ high biomarker staining have significantly increased survival relative to patients with PTPµ low biomarker staining. The last outcome recorded for a patient (i.e., living or deceased) at the end of the follow up period was carried forward to generate these survival plots. The survival of all glioma patients with PTPµ high and PTPµ low is plotted either unadjusted (Figure 2a) or adjusted (Figure 2b) by gender, grade, age group, and IDH1 mutation status. Median survival times are shown below each plot. As shown in Figure 2a, the median survival of all glioma patients with PTPµ low was 13.3 months compared to 57.8 months for those with PTPµ high. After adjusting for sex, tumor grade, age group, and IDH1 mutation status, the median survival of all patients with PTPµ low was about half as long, 18.6 months, as those with PTPµ high staining, where the median survival was 38.2 months (Figure 2b).

**Figure 3:**
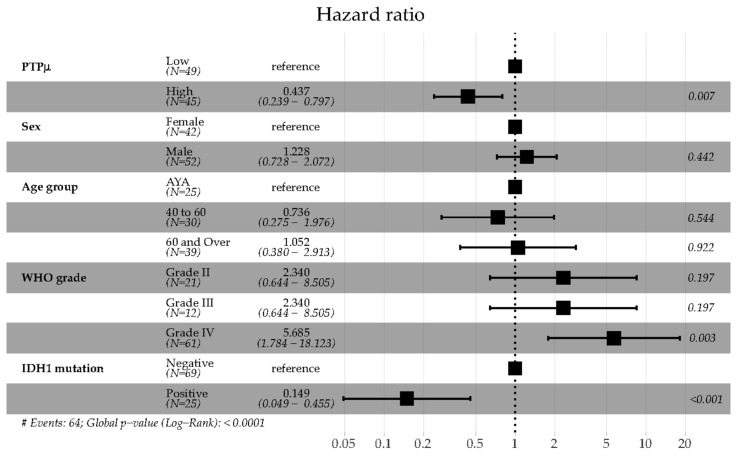
Forest plot of hazard ratios (with 95% CIs) from the final multivariable Cox proportional hazards regression model for overall survival for all glioma patients. Wald test *p*-values are shown.

Multivariable Cox proportional hazards regression survival models were generated to investigate the effects of PTPµ, sex, age, grade, IDH1 mutation status, and other parameters on overall survival. Results of the final model are summarized in the Forest Plot shown in Figure 3**.** Sex, age, WHO tumor grade, and IDH1 mutation status were all included in the final model since all four characteristics are well validated prognostic factors in glioma as mentioned above [4,5,6,22]. PTPµ high staining resulted in a significantly decreased hazard compared to PTPµ low staining (Figure 3). Males showed a slightly increased hazard compared to females, but this difference was not significant. Similarly, there were no significant differences in the hazard of death among patients in the different age groups. Patients with grade IV tumors had significantly increased hazard ratios relative to patients with lower grade tumors. Consistent with previous studies, patients with mutant IDH1 had a significantly reduced hazard of death relative to wild-type IDH1 glioma patients (Figure 3).

**Figure 4:**
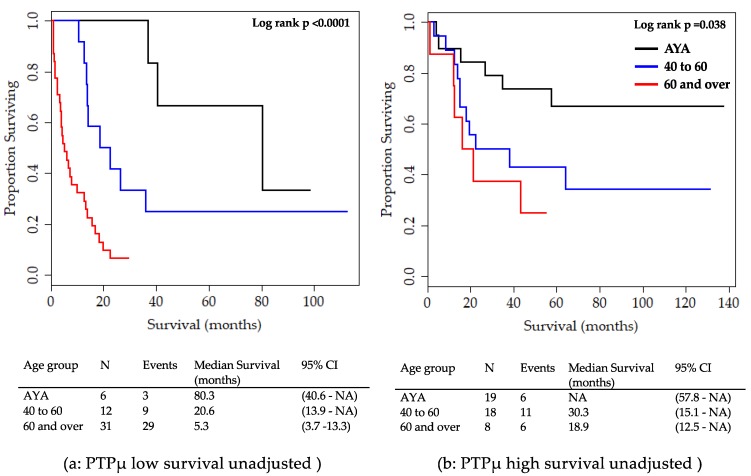
Kaplan Meier survival plots for overall survival by PTPµ low versus high staining and age at diagnosis for all glioma patients. (**a**) Unadjusted survival for PTPµ low patients. (**b**) Unadjusted survival for PTPµ high patients.

To better visualize overall survival among patients with PTPµ low and PTPµ high staining in different age categories, survival data for patients in each PTPµ category were plotted, as shown in Figure 4. Unadjusted Kaplan Meier survival plots were calculated for glioma patients with PTPµ low (Figure 4a) and PTPµ high (Figure 4b). Too few patients were available in each category to make meaningful Kaplan Meier survival plots that adjusted for sex, grade, and IDH1 mutation. Of the 49 patients with PTPµ low staining, six were AYA, 12 were patients aged 40 to 60, and 31 were patients aged 60 and over. In the PTPµ low group, younger patients had longer median survival times than older patients (Figure 4a). Patients aged 60 and over with low levels of PTPµ staining had a median survival time of 5.3 months, patients 40–60 years old had a median survival of 20.6 months, and the AYA patients had an almost four-fold longer median survival of 80.3 months (Figure 4a).

The distribution of age groups was different for patients with PTPµ high staining; of the 45 high, 19 were AYA, 18 were aged 40 to 60, and only eight were aged 60 and over. As with PTPµ low biomarker staining, the unadjusted overall survival for patients in the 60 and over group was worse than that of the other two age categories for the PTPµ high biomarker (Figure 4b). AYA patients with high levels of PTPµ had longer survival compared to the other the age groups (Figure 4b), but median survival time could not be determined because only six deaths were recorded among the 19 AYA patients.

Comparison of PTPµ low and PTPµ high biomarker staining within a given age group reveals some interesting observations (Figure 4). For instance, patients in the oldest age group with PTPµ high staining had a significantly longer median overall survival of 18.9 months (Figure 4b) compared to 5.3 months for those patients 60 and over in the PTPµ low group (Figure 4a; log rank *p*-value = 0.025). The trend was similar although not statistically significant for the other two age groups. In the 40 to 60 age category, the median survival was 30.3 months for the PTPµ high versus 20.6 months for PTPµ low patients. The median survival time for AYA patients with PTPµ high could not be determined and cannot be compared to that of AYA patients with PTPµ low in this study due to the length of the follow-up period. Of note, 13 of 19 PTPµ high AYA patients survived through the follow-up period compared to three of six in the PTPµ low group.

**Figure 5:**
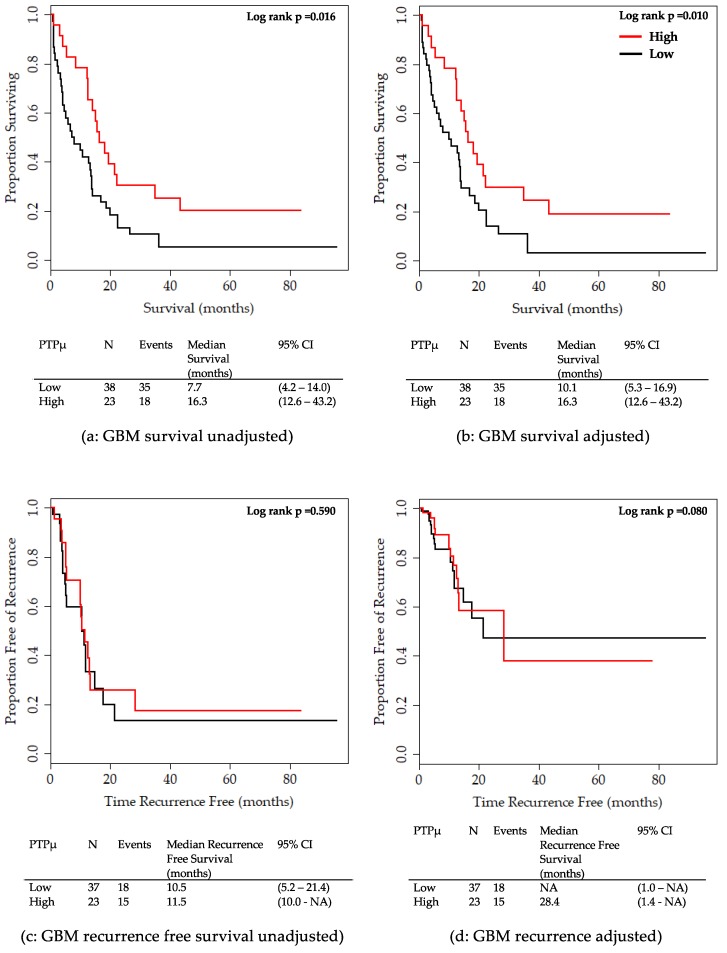
Kaplan Meier survival plots for overall survival and recurrence-free survival by PTPµ high versus PTPµ low staining for GBM patients only. (**a**) Unadjusted overall survival for GBM patients. (**b**) Overall survival adjusted for sex, age group, and IDH1 mutation for GBM patients. (**c**) Unadjusted recurrence-free survival for GBM patients. (**d**) Overall recurrence-free survival adjusted for sex, age group, and IDH1 mutation for GBM patients. Median overall survival or recurrence-free survival with 95% CIs are shown below each plot. Based on the log-rank test, recurrence-free survival curves were not significantly different between PTPµ high and low patients for either unadjusted or adjusted curves.

Next, the 61 patients with GBM were analyzed separately to better examine the relationship between survival and PTPµ staining in these patients. Kaplan Meier survival plots for overall survival are shown unadjusted or adjusted for sex, age group, and IDH1 mutation status (Figure 5). GBM patients with PTPµ high staining showed significantly better survival compared to those with PTPµ low staining in both unadjusted (Figure 5a) and adjusted plots (Figure 5b). In contrast, no significant differences were detected between GBM patients with PTPµ low and PTPµ high staining in terms of recurrence-free survival (Figure 5c,d).

**Figure 6:**
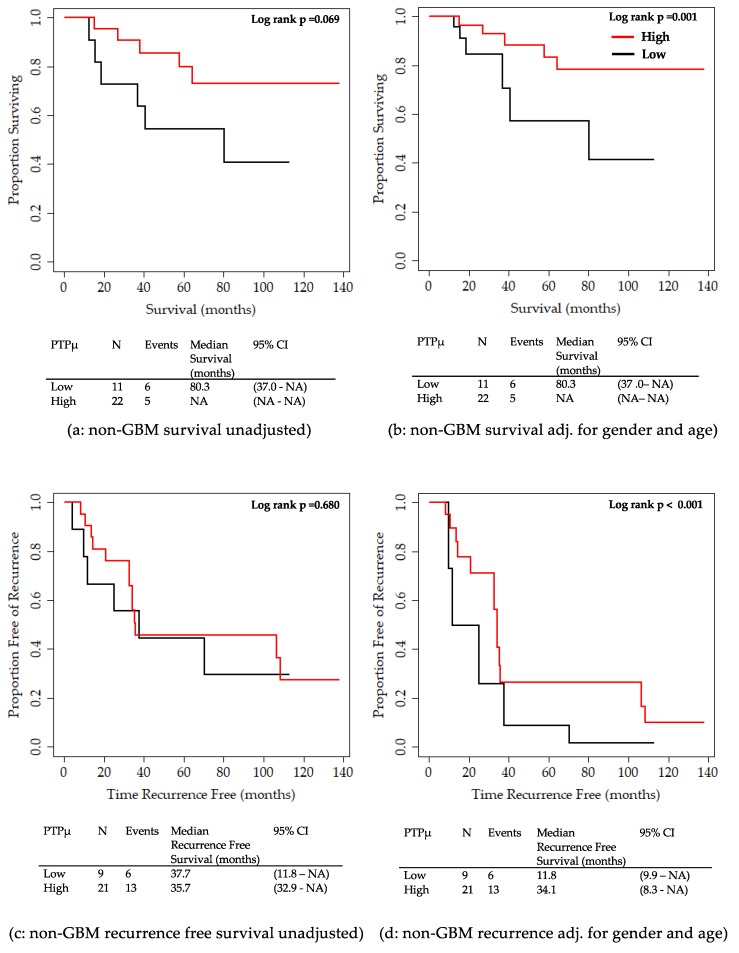
Kaplan Meier survival plots for overall survival and recurrence-free survival by PTPµ high versus PTPµ low staining for patients with lower grade gliomas. (**a**) Unadjusted overall survival for lower grade (non-GBM) patients. (**b**) Overall survival adjusted for sex, age group, and IDH1 mutation for non-GBM patients. (**c**) Unadjusted recurrence-free survival for non-GBM patients. (**d**) Overall recurrence-free survival adjusted for sex, age group, and IDH1 mutation for non-GBM patients. Median overall survival or recurrence-free survival with 95% CIs are shown below each plot. Based on the log-rank test, the adjusted recurrence-free survival curves were significantly different between PTPµ high and low non-GBM patients.

Finally, we examined the 33 remaining patients with lower grade gliomas (non-GBM), including astrocytomas (grade II and III), oligoastrocytomas, and oligodendrogliomas, to determine whether PTPµ staining correlated with overall survival (Figure 6a,b) or recurrence-free survival (Figure 6c,d). As with GBM patients, patients with lower grade tumors but PTPµ high levels had longer overall survival than those with PTPµ low levels, although this difference was only significant after adjusting for sex, age group, and IDH1 mutation status (Figure 6b). There was no difference in the unadjusted recurrence-free survival for glioma patients with non-GBM tumors with high and low PTPµ biomarker staining (Figure 6c). However, after adjusting for sex, age group, and IDH1 mutation status, the PTPµ high non-GBM glioma patients had significantly longer recurrence free survival times than the PTPµ low non-GBM patients, 34.1 versus 11.8 months, respectively (Figure 6d).

## 3. Discussion

The most recent WHO Classification of Tumors of the Central Nervous System recommendations combine basic histology with either immunohistochemical or genetic tests for mutated IDH1 status, transcriptional regulator (ATRX) loss, and TP53 mutation or 1p/19q chromosomal deletion status to differentiate tumors [22]. Using these molecular markers, gliomas can be more accurately classified as diffuse astrocytoma, oligodendroglioma, oligoastrocytoma, or the glioma with the worst overall prognosis, GBM [22]. Of particular interest was the recommendation that molecular data and genotype overrule histology when discordant results arise [22].

Sequencing studies by The Cancer Genome Atlas (TCGA) identified the common mutation of IDH1 in GBM, with an observation that ~10% of GBM patients harbored IDH1 mutations [23]. IDH1 mutations were associated with increased overall survival of GBM patients and occurred preferentially in young patients and those with secondary GBM [23], that is GBM progressing from a lower grade glioma as opposed to GBMs that arise de novo, i.e., primary GBM. A further refinement of glioma subtypes was accepted in the 2016 WHO guidelines by adding ATRX and TP53 mutational analysis alongside evaluation of 1p/19q chromosomal co-deletion [22]. GBMs and astrocytomas are classified as IDH mutant or wild-type [24]. Oligodendrogliomas can be distinguished from astrocytomas based on ATRX and TP53 mutations (observed in astrocytomas only) versus 1p/19q co-deletion (observed in oligodendrogliomas only along with IDH1 mutation) [24]. The use of immunohistochemistry for both IDH1 and ATRX mutation analysis should simplify the adoption of molecular diagnostics in the neurohistological setting [25]. Based on molecular findings, new predictions for disease outcome can also be determined. For example, the presence of IDH1 mutations and 1p/19q co-deletions are associated with better survival outcomes for grade II, III, and IV gliomas [7,23,26,27], which may be relevant for determining treatment options for lower grade glioma patients with worse prognoses [7,26].

The data presented here suggest that high levels of PTPµ staining correlate with longer overall survival (anywhere from one and a half to three times longer) for patients of similar age. Since high PTPµ staining is correlated with improved survival of all age groups, the PTPµ biomarker may be an important prognostic marker. Unlike the markers discussed above, the changes observed in PTPµ in glioma are all post-translational in nature and not at the level of DNA. There is little evidence of PTPµ changes at either the DNA or the RNA level in brain tumors in the literature. The TCGA database indicates 13 mutations in the PTPµ gene (PTPRM) coding region, most of them low impact mutations. We previously observed differences in full-length PTPµ and proteolytic fragments of PTPµ in different glioma types, including GBM by immunoblot [17]. When full-length PTPµ protein was added back to the invasive glioma LN-229 cell line, a cell line characterized by low amounts of full length PTPµ and high amounts of PTPµ fragments, cell migration was reduced [15]. We found that PTPµ fragment expression was essential for promoting cell migration and cell survival in this cell line [15]. Based on these results, we hypothesized that proteolytic cleavage of PTPµ impacts adhesion between adjacent cells, leading to a loss of contact inhibition of growth and promotion of cancer cell migration and invasion [28].

PTPµ high biomarker staining and IDH1 mutation both substantially reduced the hazard ratio of death, as shown in Figure 3. Additional studies with more patients in each age group are needed to determine whether these biomarkers are involved in one or more common pathways leading to oncogenesis and/or prolonged survival.

Current practice is to utilize Clinical Laboratory Improvements Amendments (CLIA)-approved and commercially available monoclonal antibodies (mAbs) for the most common mutation of IDH1 and ATRX for routine grading of gliomas [25]. If validated by additional studies, the SBK4-TR agent could be used in a similar setting and would allow quick and convenient one-step staining, as the Texas Red fluorophore is already conjugated to the SBK4 peptide. In future studies, we will use this same reagent to validate our results in an independent dataset of patient tissue.

In addition to using the SBK agents to predict patient outcomes, these agents could also be used in fluorescence-guided surgical resection of glioma [9] for patients whose biopsy is positive for the PTPµ biomarker. 5-ALA is currently approved to be used in GBM surgery as it distinguishes tumor tissue from normal tissue by the preferential conversion of 5-ALA to fluorescent porphyrins (PpIX) in the heme biosynthesis cycle, which occurs at a higher rate in epithelial and tumor tissue [29]. PpIX fluoresces under 400–410 nm wavelength excitation and emits at 635–705 nm and can be visualized with a fluorescent surgical microscope. It is very effective at delineating the main GBM tissue mass, with 92% positive predictive value, 77% specificity, and 79% negative predictive value [12], and was recently approved by the U.S. FDA for this purpose. However, it is less effective at labeling the dispersive GBM tumor border [13,30]. The use of 5-ALA in the surgical resection of GBM results in a significant improvement in the extent of tumor resection (65% versus 36% with white light alone) and yields an improvement in six month progression-free survival [11]. From previous studies, we know that the SBK agents are very effective at labeling the main tumor mass and the tumor’s invasive edge [19]. The SBK agents can be conjugated to any fluorophore, including those in the near-infrared range to minimize tissue interference. Since the PTPµ biomarker signifies cancer and the data presented demonstrate that the SBK4-TR agent labels both low- and high-grade glioma tissue, SBK4-TR could be useful for fluorescence-guided surgical resection of gliomas, either alone or multiplexed with 5-ALA for double labeling.

Brain tumors are the third most common malignancy in AYA patients between 15 and 39 years old [31]. Until recently, AYA populations have been lumped with either pediatric or adult patients, and their treatment has varied between following pediatric or adult guidelines, neither of which may be appropriate for this disease [32]. With the advent of molecular characterization of malignancy, fundamental differences between AYA patients and other age groups have been identified, clarifying that separate disease mechanisms are at play. In the case of glioma, pediatric, AYA, and adult patients have molecular distinctions between and within different glioma grades [32]. In GBM, for example, TP53 and IDH1 mutations and phosphatase and tensin homolog (PTEN) deletion are frequently observed in patients under 40 [23,33], as is hypermethylation of the CpG island methylator (C-GIMP) phenotype [34]. All are correlated with better prognosis. In this data set, there are higher levels of the PTPµ biomarker in AYA patients and in the 40 to 60 adult glioma patients, while patients 60 years and over tend to have low PTPµ staining.

In older GBM patients, epidermal growth factor receptor (EGFR) amplification and PTEN deletions are observed in a majority of cases, and IDH1 mutations are rarely observed [33]. Of note, our data included a subset of GBM patients over 60 with PTPµ high staining and median survival times more than three times that of GBM patients in the same age group with PTPµ low staining. As with the IDH1 mutation, PTPµ high staining correlates with improved survival. Unlike IDH1 mutation status, the PTPµ biomarker may be a relevant prognosis marker for all age groups. In summary, the data presented here indicate the exciting possibility that the staining of the PTPµ biomarker may be used to predict clinical outcomes of glioma patients.

## 4. Materials and Methods

### 4.1. Study Ethics and Patient Information

Glioma patients were identified and prospectively consented to the Ohio Brain Tumor Study (OBTS, PIs: Barnholtz-Sloan and Sloan) under approval from the University Hospitals Institutional Review Board (IRB Number CC296; Approval 24 May 2018). Clinical and pathological data were gathered for each patient and included age at diagnosis, sex, race, WHO grade, histological type, overall survival, overall vital status, recurrent status, and recurrence-free survival. The IDH1 mutation status was obtained for some patient samples through the TCGA database (21 samples) or as part of the medical record (30 samples). The remaining samples were stained for IDH1 mutation as described below. The last outcome recorded (i.e., living or recurrence-free) at the conclusion of the follow-up period was carried forward to generate the Kaplan Meier plots used to illustrate overall survival or recurrence-free survival. No recurrence data were available for four patients (three in the PTPµ low group and one in the PTPµ high group), thus these were excluded from analyses of recurrence-free survival. The mean time to recurrence shown in Table 1 for each group was calculated only from patients who experienced a recurrence.

### 4.2. Reagents

The SBK4 peptide used for tissue staining was synthesized as described [18]. The *N*-terminal glycine of SBK4 peptide was coupled to Texas Red (TR; Molecular Probes Inc, Eugene, OR, USA) as described [18] to make the fluorescent agent. Anti-IDH1 R132H Monoclonal Antibody clone H09 [American Research Products (Dianova GmbH), Waltham, MA, USA] reacts specifically with the isocitrate dehydrogenase 1 (IDH1) R132H point mutation in tissue sections from formalin-fixed brain tumor specimens.

### 4.3. Biomarker Labeling of Human Glioma Tissue

All tumor samples used for this study were obtained from the OBTS, which makes patient samples available to researchers. The OBTS generated three TMAs to facilitate screening a large number of patient biopsy tissues, and these were stained for the PTPµ biomarker. To supplement these samples, additional individual biopsy samples were also screened. Together, these TMAs and slides represented samples from 94 glioma patients (25 adolescent and young adult and 69 adult) with astrocytomas (*n* = 12), oligodendroglioma (*n* = 14), oligoastrocytoma (*n* = 7), and GBM (*n* = 61). Tissue staining with SBK4-TR was described [18]. Positive controls (GBM) and negative controls (Epilepsy) were tested with the TMAs or individual slides. Tumor samples were obtained formalin-fixed and paraffin-embedded. Prior to staining, the TMAs or slides were deparaffinized and blocked with 2% goat serum in phosphate buffered saline (PBS) for 20 min at room temperature (RT). The samples were then incubated with SBK4-TR agent diluted in 2% goat serum in PBS at RT for 1 hr in the dark. Following a PBS rinse, the TMAs or slides were coverslipped with Vectashield Hard Set Mounting Medium (Vector Laboratories, Inc., Burlingame, CA, USA) and imaged on a Hamamatsu Nanozoomer S60 slide scanner (Bridgewater, NJ, USA). Some samples were also stained for the IDH1 mutation. For IDH1 staining, antigen retrieval was performed in a citrate buffer. Antibody binding was detected using MACH4 horseradish peroxidase (Biocare, Pacheco, CA, USA), and diaminobenzidine was used as a chromogenic substrate. The sections were counterstained with hematoxylin and eosin (H&E) and mounted with Ecomount (Biocare, Pacheco, CA, USA) and imaged.

Tissue staining for both PTPµ and IDH1 biomarkers was quantified by blinded observers. For PTPµ, an initial scoring system of one to four was used to capture staining intensity information about the samples with a staining level of one indicating low fluorescence and level four indicating high fluorescence. After reviewing all of the results, the PTPµ biomarker was dichotomized as either low or high to better reflect the biphasic nature of the staining pattern. For IDH1, samples were scored as either positive for the mutation or negative as it is done clinically.

### 4.4. Statistical Analysis

Data were analyzed using version 3.5.1 of the R software. Summaries of PTPµ staining in comparison to the indicated clinicopathological characteristics were performed using the “tableone” package. Numbers and percentages of categorical variables were compared using the Chi square test. For continuous variables, means and standard deviations were calculated and compared using a t test. Survival analyses were performed using the “survival” and “survminer” packages in R. The Kaplan Meier method and log-rank tests were used for generating unadjusted survival curves and testing for significance as indicated. Multivariable models using Cox proportional hazards regression were generated to incorporate the possible contribution of additional clinicopathological features to overall survival. The final model selected for all patient data adjusted for sex, age group, tumor grade, and IDH1 mutation. The global log-rank *p*-values are shown for the survival curves with the three age groups indicated. In all cases, *p*-values <0.05 were considered statistically significant.

## Figures and Tables

**Table 1:** Clinicopathological characteristics of glioma patients from the Ohio Brain Tumor Study with low and high staining for the PTPµ biomarker. The recurrence times indicated represent the mean recurrence times only for patients who had a recurrence. The mean survival times provided are for all patients combined.

Variable	Category	PTPµ Low	PTPµ High	*p* Test
Number		49	45	
Sex (%)	Female	17 (34.7)	25 (55.6)	0.068 ^1^
	Male	32 (65.3)	20 (44.4)	
Race (%)	Asian	1 (2.0)	0 (0.0)	0.546 ^1^
	Black	2 (4.1)	1 (2.2)	
	White	46 (93.9)	44 (97.8)	
Age at Diagnosis [mean (sd)]		62.2 (14.4)	46.3 (15.5)	<0.001 ^2^
Histologic Type (%)	Astrocytoma	3 (6.1)	9 (20.0)	0.044 ^1^
	Glioblastoma	38 (77.6)	23 (51.1)	
	Oligoastrocytoma	2 (4.1)	5 (11.1)	
	Oligodendroglioma	6 (12.2)	8 (17.8)	
WHO Grade (%)	Grade II	5 (10.2)	16 (35.6)	0.010 ^1^
	Grade III	6 (12.2)	6 (13.3)	
	Grade IV	38 (77.6)	23 (51.1)	
Recurrence Status (%)	No	22 (47.8)	16 (36.4)	0.375 ^1^
	Yes	24 (52.2)	28 (63.6)	
Recurrence time in months [mean (sd)]		12.8 (14.9)	22.6 (26.4)	0.114 ^2^
Survival Status (%)	Alive	8 (16.3)	22 (48.9)	0.002 ^1^
	Deceased	41 (83.7)	23 (51.1)	
Survival time in months [mean (sd)]		22.4 (27.9)	48 (38.1)	<0.001 ^2^
Age Group (%)	AYA ^3^	6 (12.2)	19 (42.2)	<0.001 ^1^
	40 to 60	12 (24.5)	18 (40.0)	
	60 and over	31 (63.3)	8 (17.8)	
IDH1 mutation (%)	Negative	41 (83.7)	28 (62.2)	0.034 ^1^
	Positive	8 (16.3)	17 (37.8)	

^1^*p*-value from Chi-Square test; ^2^*p*-value from t test; ^3^ Adolescent and Young Adult.

## Abbreviations

5-ALA	5-aminolevulinic acid
ATRX	Transcriptional regulator ATRX
AYA	Adolescent and Young Adult
C-GIMP	CpG island Methylator Phenotype
CLIA	Clinical Laboratory Improvements Amendments
DOTA	1,4,7,10-Tetraazacyclododecane-1,4,7,10-tetraacetic acid
H&E	Hematoxylin and Eosin
IDH	Isocitrate Dehydrogenase
mAbs	monoclonal Antibodies
MRI	Magnetic Resonance Imaging
GBM	Glioblastoma
RT	Room Temperature
TMA	Tissue Microarray
TR	Texas Red
WHO	World Health Organization
